# A Case of Vesico Urethro-Vaginal Fistula, the Result of Venereal Ulceration

**Published:** 1877-07

**Authors:** A. L. Galabin


A Case of Vesico-Urethro-Vaginal Fistula, the Result of Venereal Ulceration.—*By A. L. Galabin, M.A., M.D.*—The patient, Elizabeth F., twenty-six years of age, single, a domestic servant, came first among my out-patients in October, 1876. She complained of incontinence of urine, and stated that it had come on gradually about six months previously, being felt at first only while she was walking or standing, continence remaining perfect in the recumbent position. Lately, however, incontinence had been complete. No history of any kind could be elicited to account for it. The vulva and surrounding parts were much inflamed and excoriated, the nymphæ irregular, and the hymen not present. On examination by speculum, the os uteri was found to be abraded. The urine was alkaline, and contained phosphatic deposits. Being in doubt whether the local inflammation could be the result simply of the irritation set up by the urine, or was due to some venereal affection, I sent the patient for the opinion of one of my surgical colleagues. He reported that he found no positive proof of any venereal causation, and considered that the inflammation might be the result of the incontinence of urine, which was possibly connected with the presence of a sensitive vascular caruncle, which existed at the orific of the urethra.

The patient was accordingly admitted into the obstetric ward on November 8th, 1876; the urethral caruncle was destroyed by means of the benzoline cautery, and strong nitric acid was applied to the abraded cervix. A few days later the existence of syphilis became manifest. The excoriations around the vulva and anus acquired the distinct character of mucous tubercles; the throat was sore, and a superficial ulceration was discovered on the left tonsil; several spots of scaly syphilitic rash also appeared about the face. The patient was then subjected to mercurial treatment until December 6th, when it had to be discontinued for a time on account of salivation. The mucous tubercles were dusted with calomel powder, and to the excoriations a solution of nitrate of silver, of the strength of ten grains to the ounce, was applied, with the effect of much improving their condition. The incontinence of urine continuing, a further examination was made on January 22d. It was then discovered that not only had the urethral caruncle disappeared, but the urethra had become completely occluded near its orifice from the effect of the cautery. It was thus evident that a fistula must exist, and a careful digital exploration of the vagina was therefore made, which had hitherto not been done, on account of the presence of syphilis. It was found that at three quarters of an inch from its orifice, the urethra was drawn upwards and firmly adherent to the posterior surface of the pubes. At this point a diagonal fissure existed, which at first appeared to involve the urethra only. A bent probe was passed through the fistula, and the occluded portion of the urethra divided upon its point by the knife. A catheter was afterwards passed daily, in order to prevent the occlusion again taking place. The patient now confessed, what she had previously concealed, that she had been delivered of a child nearly three years before. This could not, however, be connected with the production of the fistula, the incontinence of urine being only of about six months' duration.

On February 12th, seven days after the end of a menstrual period, it was decided to undertake the operation for cure of the fistula, although the general condition of the patient was by no means fully satisfactory, the manifestations of syphilis not having altogether disappeared, and the urine being still alkaline, with a phosphatic deposit. She had proved incapable of bearing any continuous administration of mercury, but iodide of potassium, in ten-grain doses three times a day, had been given for a week prior to the operation, and was continued afterwards. After trial of the left semi-prone position with Sims' speculum, it was found that this did not bring the fistula so well into view as Simon's exaggerated lithotomy position *(Steiss-ruckenlage)* with the buttocks raised high, and the thighs strongly flexed. The latter was accordingly chosen for the operation. A trial was made of drawing down the cervix externally, as recommended by Simon, but although this could be done without much force, the effect was that the fistula still remained drawn up behind the pubes, and was overlapped by a fold of mucous membrane. The plan was therefore adopted of fixing a double tenaculum forceps near the posterior angle of the fistula, and by this means drawing it slightly downwards. No speculum was used, but the perineum was held back by a large two-pronged surgical retractor, such as are used in amputations. The incisions were made entirely by the knife, and since only half the length of the urethra remained in front of the fistula, and there was, therefore, fear that continence might not be restored, even though the fistula were closed, care was taken to remove the least possible amount of tissue in vivifying the anterior margin, a result which was much facilitated by Simon's position. The fissure previously felt proved to be the entrance of a funnel-shaped opening expanding upwards, lined by cicatricial tissue, and involving all the neck of the bladder. When, therefore, all the cicatricial tissue had been removed, the finger passed freely into the bladder. After somewhat free hemorrhage had been checked by iced water, the margins were united over a gum elastic catheter by five silver sutures, which were simply twisted, the line of union being diagonal. The catheter was tied in and connected by an india-rubber tube with a vessel beneath the bed. It was left in place for three days, the bladder being washed out daily, but after that the patient was allowed to pass her urine at pleasure.

There were some signs of bladder irritation for the first few days, and on the 16th, four days after the operation, rather serious symptoms of peritonitis commenced, the pulse rising to 124, and the temperature to 103°.4, while the abdomen was tympanitic and tongue dry. On the 21st the pain and tenderness were subsiding, and on the 22d a menstrual period came on about a week before the expected time, after which she gradually improved. The sutures were removed on the 26th, fourteen days after the operation, having been left somewhat longer on account of the peritonitis. No urine had escaped in the meantime, and the fistula was found perfectly closed. A few drops of urine escaped in the standing position for a few days after the patient first got up, but when she left the hospital on March 14th, continence was perfect.

The foregoing case may be of interest from the fact that the union was not hindered either from the constitutional syphilis, which was still giving evidence of its presence in the shape of mucous tubercles around the labia, from the alkaline condition of the urine, or from the severe febrile condition which followed the operation. The mode of origin of the fistula could not be exactly ascertained. It appeared, however, that it could not have been due to the delivery nearly three years before, although some cicatricial tissue might have been left which afterwards became the seat of the ulceration. But whether the lesion was due to a venereal but non-syphilitic sore, to the chancre which communicated the syphilis, or to tertiary syphilitic ulceration, remained uncertain. The last alternative, however, would seem to be almost excluded by the character of the symptoms, which was that of the earlier constitutional manifestations of the disease.

As to the method of performing the operation, I am disposed to think that, in cases of urethro-vaginal fistula, in which union is more difficult to secure, on account of the thinness of the septum from which the margins have to be cut, and in which it is desirable to attain the utmost possible economy of tissue, in order to secure complete continence after closure of the fistula, the position of Simon has often the advantage over that of Sims'. As a testimony to the improvement which has taken place in English obstetrics of late years with regard to an earlier interference in protracted labor, I may add that all the cases of vesico-vaginal fistula admitted into the obstetric ward of Guy's Hospital within the last three years have been cases either of old fistulæ, of from six to ten years' standing, most of which had been previously operated on without complete success, or of fistulæ the result either of venereal ulceration, of attempted operation for atresia vaginæ, or of the impaction of a calculus in the urethra. Not one had been the consequence of recent delivery.—*Obs. Jour.*

